# Impact of vertical stratification on the 2020 spring bloom in the Yellow Sea

**DOI:** 10.1038/s41598-023-40503-z

**Published:** 2023-08-31

**Authors:** Go-Un Kim, Jaeik Lee, Yong Sun Kim, Jae Hoon Noh, Young Shin Kwon, Howon Lee, Meehye Lee, Jongmin Jeong, Myung Jin Hyun, Jongseok Won, Jin-Yong Jeong

**Affiliations:** 1 Korea Institute of Ocean Science and Technology, Busan, South Korea; 2 Ocean Science and Technology School, Korea Maritime and Ocean University, Busan, South Korea; 3 Department of Earth and Environmental Sciences, Korea University, Seoul, South Korea; 4 Department of Ocean Science, University of Science and Technology, Daejeon, South Korea

**Keywords:** Ocean sciences, Physical oceanography, Environmental impact, Physical oceanography

## Abstract

The Yellow Sea is one of the world’s most abundant marine resources, providing food and economic benefits to the Korean and Chinese populations. In spring 2020, a decrease in the intensity of phytoplankton bloom was observed. While one study attributed this decline to a decrease in nutrient associated with the COVID-19 pandemic, our previous research proposed weakened thermal stratification accompanied by a surface cooling anomaly as the cause. However, the relationship between the marine environment and ecosystem has not been fully elucidated. Using observations and marine physical-biogeochemical model data, we identified the weakened stratification as a critical factor for suppressing the 2020 spring bloom. Intense vertical mixing hindered the accumulation of nutrient and chlorophyll-a concentrations within the euphotic zone, resulting in a diminished phytoplankton bloom. In contrast, reduced nitrate and phosphate concentrations in 2020 were insignificant compared to those in 2017–2019, despite the notable decline in PM_2.5_ in March 2020 due to COVID-19. In April 2020, nutrient levels fell within the range of interannual variability based on long-term observations, reflecting a negligible effect on the spring phytoplankton bloom. Our findings provide insight into the importance of marine physical factors on the phytoplankton biomass in the Yellow Sea.

## Introduction

The Yellow Sea, designated as Large Marine Ecosystem 48, is one of the most productive marine fisheries worldwide (https://www.lmehub.net/#yellow-sea)^1,2^. It has the geographical characteristics of a shallow continental shelf sea with an average depth of 44 m and a semi-enclosed marginal sea surrounded by China and the Korean peninsula. Its marine environment is greatly influenced by air-sea interaction processes, particularly the seasonal variation of the East Asian monsoon winds that alternate between cold and dry winds from the northwest and warm and moist winds from the southeast, and the northward intrusion of the Yellow Sea Warm Current as an up-wind flow from the Kuroshio associated with winter monsoon wind^3,4^. A comprehensive understanding of the Yellow Sea environment is therefore essential, as it affects marine ecosystems and further people’s lives near the Yellow Sea region of Korea and China.

In temperate zones, the factors limiting phytoplankton biomass in marine ecosystems depend not only on light and nutrient availability but also on other environmental conditions with seasonal variation, such as vertical mixing, water temperature, salinity, aerosol deposition, and estuaries^5–13^. The water column of the Yellow Sea is fully mixed from November to early April, resulting in uniform distribution of nutrients and chlorophyll-a concentrations^6,14^. With the onset of spring, solar radiation and water temperatures increase, triggering the formation of thermocline that retains trapping nutrients and chlorophyll-a within the sunlight zone^5,10,12^. The warm current into the Yellow Sea serves as a source of nutrients during winter and early spring^11^, whereas spring Asian dust events and the riverine waters from the Yangtze River during the summer monsoon period provide additional nutrients to the surface of the Yellow Sea^8,9,11^.

During the spring of 2020, the Yellow Sea experienced a substantial suppression of phytoplankton bloom, with a reduction in the bloom intensity by approximately 30% compared to the average bloom observed from 2015 to 2019^15,16^. Previous studies have proposed different explanations for this decrease: weakened thermal stratification^16^ and reduced nutrient level^15^. According to Yoon et al.^15^, anthropogenic air pollutant emissions were reduced from February to March as the Chinese government imposed a total lockdown from 23 January to 7 April 2020 owing to the COVID-19 pandemic^15,17^. Since atmospheric nitrogen deposition from northern China accounts for approximately 70% of the total new nitrogen inputs^18^, the nutrient supply to the Yellow Sea in spring 2020 was expected to decrease along with the reduction in anthropogenic pollutant emissions. The resultant reduced nutrients might have caused the decline in phytoplankton biomass over the Yellow Sea rather than variations in marine physical variables, including irradiance, vertical mixing, and river discharges. In contrast, Kim et al.^16^ argued that weakened stratification associated with the anomalous cold surface could suppress and delay the phytoplankton bloom in the Yellow Sea by analyzing the observed temperature and PM2.5 concentration in tandem with numerical experiments based on a simple 1-D turbulence model. The water temperature was 1.2 °C higher from January to March, but 1.0 °C lower in May 2020 than those of the years 2017–2019, as the extreme cold lasted around April across Northeast Asia^19,20^. The resultant relatively cold water in the upper layer was accompanied by weak stratification in the spring of 2020 compared to that in 2017–2019. However, there is a lack of observational data analysis, such as vertical stratification and nutrient levels, and previous numerical experiments did not consider the marine ecosystem. As a result, a complete understanding of the relationship between marine environmental factors and phytoplankton populations during spring 2020 remains elusive.

As a follow-up to the study by Kim et al.^16^, the present study aimed to identify the factors of thermal stratification and nutrients that contributed to the suppression of phytoplankton biomass during the 2020 spring bloom in the Yellow Sea. For this purpose, we analyzed the daily observational data of physical, chemical, and biological variables in the Yellow Sea during the spring of 2017–2020 and performed numerical experiments using the Generalized Ocean Turbulence Model coupled with the biological module of European Regional Seas Ecosystem Model (GOTM-ERSEM, see Methods).

## Results

### Observed changes in the marine environment in the Yellow Sea during spring 2020

**Figure 1:**
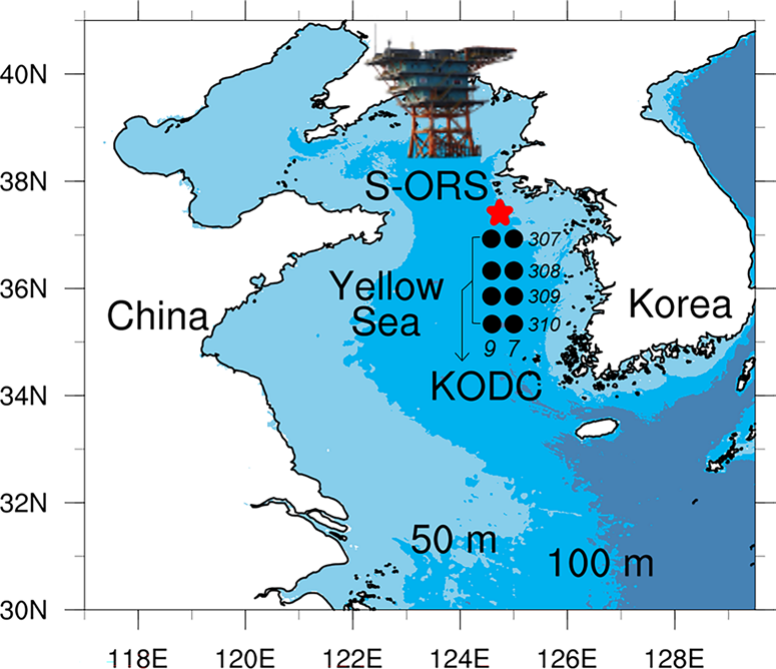
A map of the monitoring stations in the Yellow Sea with a bathymetry of 50 and 100 m. The star marker denotes the Socheongcho Ocean Research Station (S-ORS) and the filled circles represent the 7 and 9 stations on the 307 and 310 lines, respectively (i.e., a total of eight stations), from the Korea Oceanographic Data Center (KODC).

**Figure 2:**
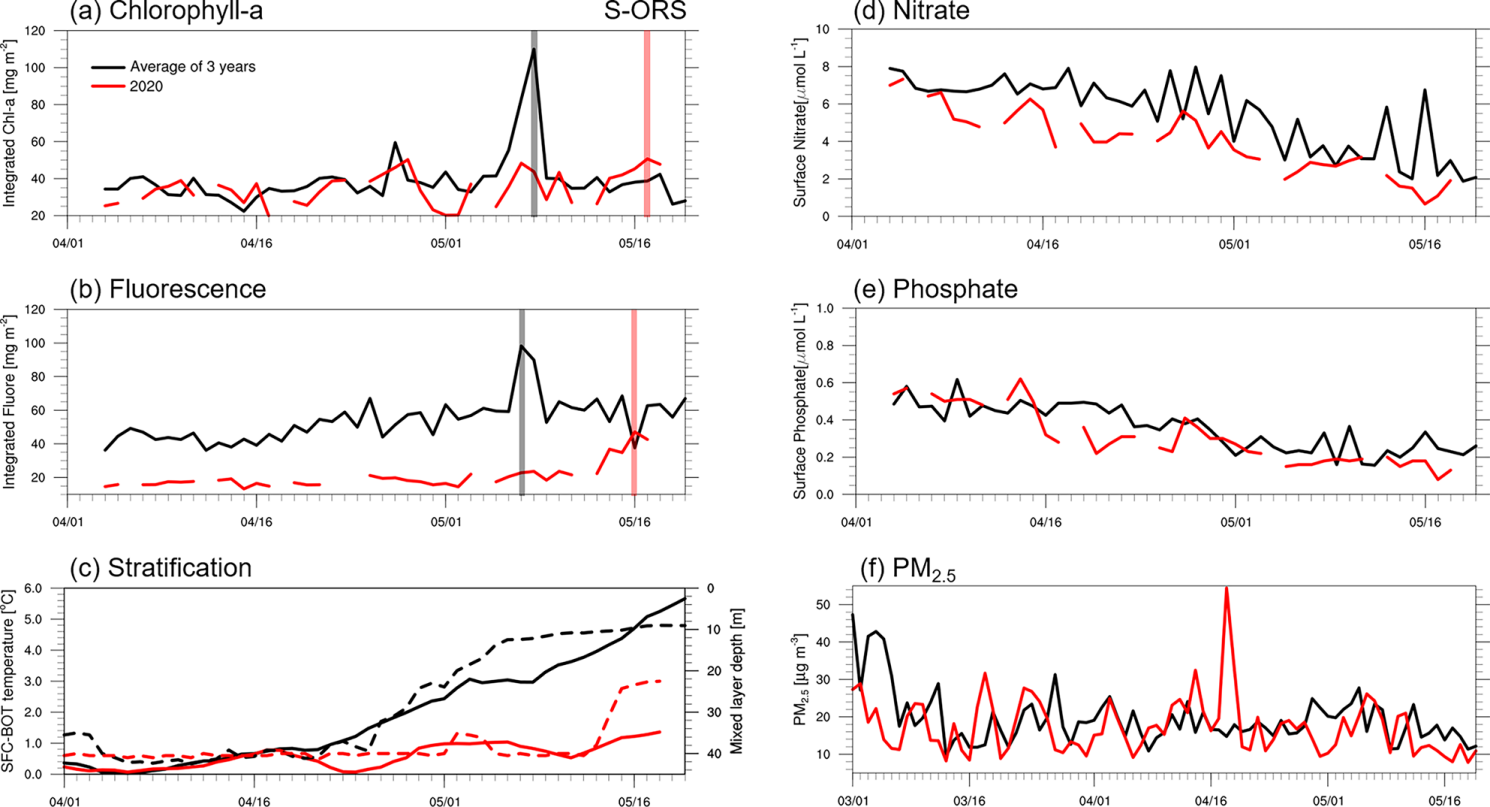
Daily time series of the (**a**) water-column integrated (0–20 m) chlorophyll-a (mg m^−2^), (**b**) integrated fluorescence (mg m^−2^), (**c**) stratification (°C), (**d**) surface nitrate (μmol L^−1^), and (**e**) surface phosphate (μmol L^−1^) from 1 April to 20 May and (**f**) PM_2.5_ (μg m^−3^) from 1 March to 20 May obtained from the S-ORS. In (**a**, **b**), the transparent lines indicate the maximum value of chlorophyll-a and fluorescence. In (**c**), the solid and dashed lines refer to the difference in the surface and bottom temperatures and the density-based mixed layer depth, respectively.

Figure 2 shows the temporal variations in chlorophyll-a concentration and fluorescence in the Socheongcho Ocean Research Station (S-ORS, see Fig. 1) from 1 April to 20 May 2017–2020. The chlorophyll-a concentration and fluorescence are commonly used indicators of phytoplankton biomass and primary productivity^21^. The integrated chlorophyll-a in the euphotic layer was the highest in late April or early May, reaching its peak value of 109.9 mg m^−2^ on 8 May for the average of 2017–2019; however, in mid-May 2020, the maximum value was 50.6 mg m^−2^ with a less distinct peak (Fig. 2a). The temporal pattern of integrated fluorescence was similar to that of the chlorophyll-a (Fig. 2b). These results suggest that the 2020 spring bloom in the Yellow Sea had approximately 50% lower intensity than that in the three preceding years, with its peak delay of approximately 1–2 weeks^16^.

To investigate the factors affecting phytoplankton biomass in the Yellow Sea during spring 2020, we examined the daily time series of vertical stratification and nutrient concentrations in the S-ORS (Fig. 2c–e). The intensity of vertical stratification was estimated by the temperature difference between the surface and bottom layers. Weaker (stronger) stratification indicates a more homogeneous (heterogeneous) water mass in the vertical structure, facilitating (hindering) vertical ocean mixing. In early April, stratification was almost absent (that is, at 0 °C) for both the 2017–2019 mean and 2020, implying strong vertical mixing of one-layer system as in winter. From mid-April onwards, the thermal stratification gradually developed for the average of 2017–2019. Thereafter, the stratification formed steadily until 20 May with an increase of 0.11 °C per day. On 20 May, during spring 2017–2019, the average temperature difference between the surface and bottom was 5.6 °C. However, in 2020, the stratification virtually disappeared on 23 and 24 April and reformed at a later stage. The difference in the vertical temperature increased by 0.02 °C per day for spring 2020 with a temperature difference of 1.3 °C in mid-May (Fig. 2c). These results indicate that the thermocline’s intensity in the northeastern Yellow Sea was distinctly weaker in 2020 than that in the 2017–2019 average. Besides, the density-based mixed layer depth, as an indicator of stratification, yielded almost the same result (Fig. 2c).

**Figure 3:**
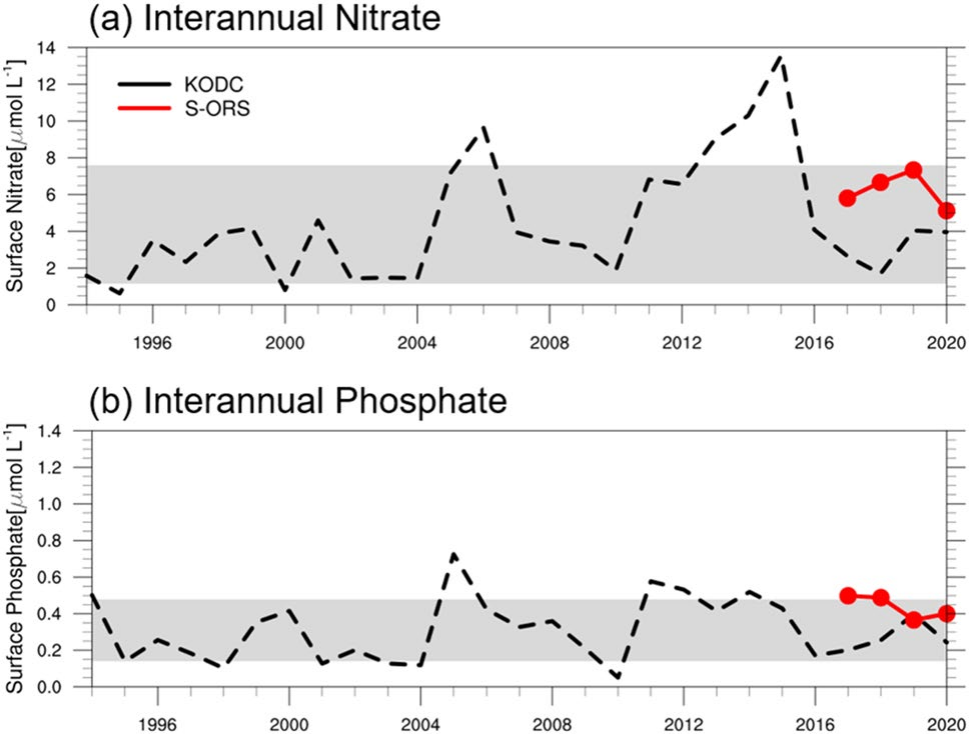
Time series of interannual variability of April (**a**) nitrate and (**b**) phosphate levels (μmol L^−1^) during the period 1994–2020 from KODC (black dashed line) and S-ORS (red solid line). Gray shaded area denotes the one standard deviation from the April climatological average between 1994 and 2020.

Regarding surface nutrient concentrations in the northeastern Yellow Sea, the differences in nitrate level between 2020 and the 2017–2019 average were minor, particularly in early April (from 6.99 to 3.11 μmol L^−1^ during 2017–2019 and from 6.27 to 1.73 μmol L^−1^ in 2020, Fig. 2d). The phosphate level in early April 2020 was slightly higher than the 2017–2019 mean (from 0.5 to 0.22 μmol L^−1^ during 2017–2019 and from 0.53 to 0.16 mol L^−1^ in 2020, Fig. 2e). To determine whether the observed nutrient concentrations from the S-ORS platform in 2020 represent a significant change, we also used nutrient data for April from the Korea Oceanographic Data Center (KODC) for a long period, from 1994 to 2020 (Fig. 3). The KODC observations were calculated by averaging eight stations in the central Yellow Sea as representative data for the offshore region like S-ORS data (Fig. 1). Over the past three decades, the interannual variability of nitrate and phosphate concentrations in April ranged from 1.13 to 7.6 μmol L^−1^ and 0.14 to 0.48 μmol L^−1^, respectively. It is worth noting that the nitrate and phosphate concentrations from the S-ORS during April 2020 (5.12 and 0.4 μmol L^−1^, respectively) fell within this range of interannual variability (Fig. 3). These results reveal that the changes in nitrate and phosphate concentrations in the Yellow Sea during 2020 were insignificant compared to those in 1994–2019, which seems to contradict the results of a previous study by Yoon et al.^15^. They reported a clear decrease in nitrate level (0–20 m) during April 2020 compared to 2015–2019, which can be attributed to reduced air pollutant emissions resulting from the COVID-19 lockdown in China. The apparent decrease in nitrate level might be owing to the inclusion of data from 2015, when the nitrate concentration was highest among the 27 years in the KODC dataset, as shown in Fig. 3a.

In particular, chlorophyll-a concentration can increase with a lag time of 1–21 days (on average 1–2 weeks) following an Asian dust event due to nutrient inputs from atmospheric deposition^9,22,23^. The dust particles, originating from natural sources such as soil, are primarily classified as part of PM_10_. However, the implementation of COVID-19 containment measures, leading to reduced human activities such as fossil fuel combustion^24^, is casually linked to a decrease in PM_2.5_ level. In March 2020, the PM_2.5_ concentration substantially decreased by 17.6 μg m^−3^ compared to 22 μg m^−3^ in the 2017–2019 average observed in S-ORS (Fig. 2f), as well as in Beijing, Wuhan, and Seoul^15,25,26^. However, nutrient concentrations in S-ORS only slightly decreased in early April 2020 compared to the three preceding years (Fig. 2d, e). This observation suggests that changes in PM_2.5_ level were unlikely to exert a distinct effect on nutrient concentrations, in contrast to the casual relationship observed between PM_10_ and chlorophyll-a concentration^9,22,23^. As the relationship between PM_2.5_ and nitrate concentration was still unclear, further research is required to fully elucidate the effects of atmospheric PM_2.5_ on the marine environment in the Yellow Sea.

### Response of chlorophyll-a concentration to stratification and nutrient changes during the spring bloom in the Yellow Sea

**Figure 4:**
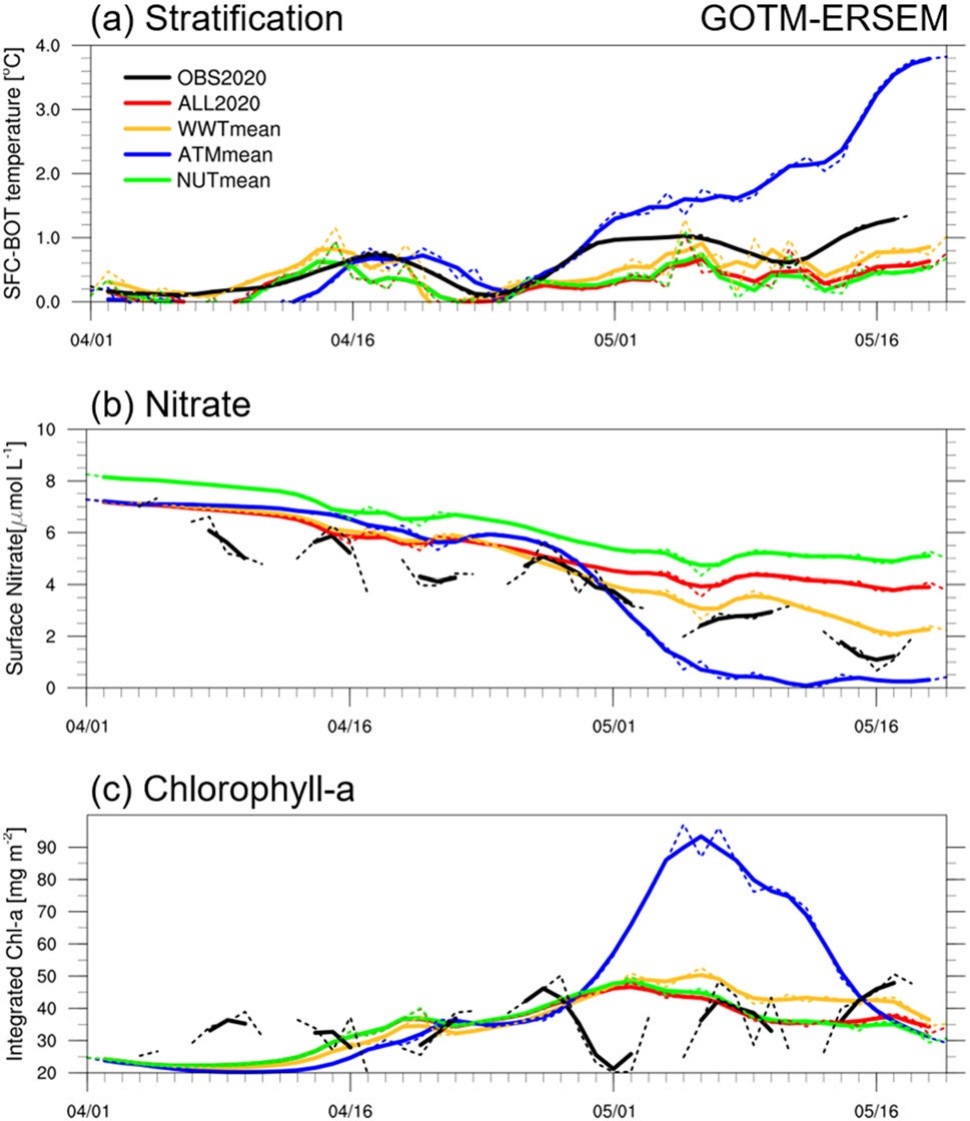
Same as Fig. 2a, c, and d in OBS2020 (black) with the addition of the GOTM-ERSEM experiments, i.e., the ALL2020 (red), WWTmean (yellow), ATMmean (blue), and NUTmean (green). The solid and dashed lines denote the 3-day running mean and one-day data, respectively.

To determine how the vertical stratification and nitrate concentration influence change in phytoplankton biomass in the Yellow Sea during the spring bloom in 2020, we performed numerical experiments using the coupled physical ecosystem model of GOTM-ERSEM and analyzed the time series of temperature and nitrate and chlorophyll-a concentration changes for four different experiments (Fig. 4). These numerical experiments included variations in initial water temperatures, nitrate concentrations, and atmospheric boundary fields (“Methods” and Table 1 for details). The focus of this study was mainly on nitrate concentration, as the difference in phosphate level between the 2020 and 2017–2019 averages was minimal and silicate is a less important nutrient for plankton growth^27^, especially compared to nitrate.

**Table 1:** List of numerical experiments.

Experiment name	Description		
	Initial water temperature	Atmospheric variables	Initial nitrate concentration
ALL2020	7.6 °C	2020	7.3 μmol L^−1^
WWTmean	2017–2019 mean (5.6 °C)	2020	7.3 μmol L^−1^
ATMmean	7.6 °C	2017–2019 mean	7.3 μmol L^−1^
NUTmean	7.6 °C	2020	2017–2019 mean (8.3 μmol L^−1^)

During the spring season, the vertical temperature distribution in the ALL2020 experiment closely resembled that of the S-ORS, with a thermal stratification rate of 0.01 °C per day in the control experiment and 0.02 °C per day in the observation (Figs. 2c, 4a, Supplementary Fig. S1a, d). The simulated spring nitrate concentration in ALL 2020 exhibited a decrease from 7 to 4 μmol L^−1^, with a relatively lower rate of nitrate consumption compared to the decrease observed from 7 to 2 μmol L^−1^ (Figs. 2d, 4b, Supplementary Fig. S1b, e). This slower decrease in nitrate consumption in ALL2020 seems to be influenced by relatively weaker stratification compared to the S-ORS. Contrarily, the column-integrated chlorophyll-a in the control case showed a time series magnitude ranging from approximately 20 to 50 mg m^−2^ throughout the period from 1 April to 20 May, which is consistent with the S-ORS observation. However, there was a discrepancy in the timing of its peak, occurring in early May in the ALL2020 and mid-May in the S-ORS (Figs. 2a, 4c, Supplementary Fig. S1c, f). In a more quantitative assessment, the standard deviation normalized root mean square error between ALL2020 and S-ORS data was 1.1 for the vertical temperature difference, 0.90 for the surface nitrate, and 1.27 for the integrated chlorophyll-a. These small error values indicate that the control experiment successfully reproduced the observation during spring 2020, even though the error value of integrated chlorophyll-a was somewhat higher owing to the discrepancies in the timing of the bloom peak. Still, the numerical model did not accurately simulate the unusual occurrence of maximum chlorophyll-a concentration near the bottom (Supplementary Fig. S1c, f). Investigating this intriguing issue is beyond the scope of our current study, which primarily focused on the euphotic layer associated with the spring bloom, and warrants further research in future studies.

Compared to ALL2020, the ATMmean experiment, which was forced with normal year (April 2017–2019) wind and air-sea heat fluxes, exhibited the most pronounced differences with an increased chlorophyll-a concentration, decreased surface nitrate level, and strong vertical stratification in spring (Fig. 4 blue lines). It is noteworthy that intense vertical stratification preceded the spring bloom, resulting in increased phytoplankton biomass within the sunlight zone and a sharp drop in nutrient concentrations at the surface. These simulated results in ATMmean are closely analogous to the 2017–2019 average observation at the S-ORS (Fig. 2a, c, d). The WWTmean experiment, characterized by strong winds and low nitrate concentration in 2020 along with the water temperature of normal years, showed a slight increase in integrated chlorophyll-a and stratification and a minor decrease in nitrate level (Fig. 4 yellow lines). In contrast, the NUTmean experiment, the same as ALL2020 except for nutrient concentration of normal years as the initial condition, exhibited decreased chlorophyll-a concentration, weak thermal stratification, and slightly reduced surface nitrate concentration, almost identical to the condition in the control experiment (Fig. 4 green lines). The experimental results indicate that the 2020 spring phytoplankton bloom was primarily controlled by the vertical stratification driven by atmospheric forcing, with initial water temperature playing a partial role and nutrient forcing having less impact.

**Figure 5:**
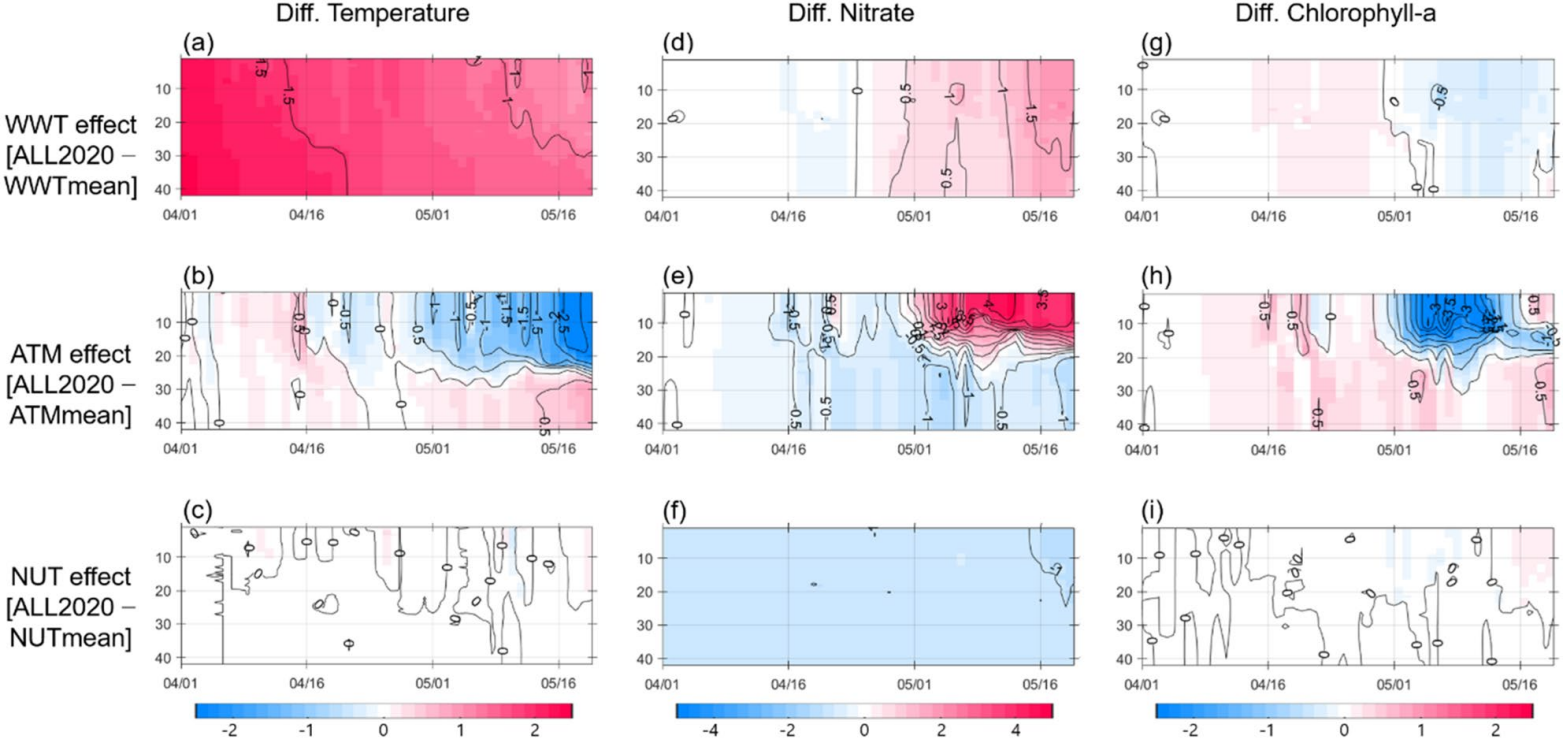
The time-depth difference in temperature (°C), nitrate concentration (μmol L^−1^), and chlorophyll-a concentration (mg m^−3^) between (**a**, **d**, **g**) ALL2020 and WWTmean, (**b**, **e**, **h**) ALL2020 and ATMmean, and (**c**, **f**, **i**) ALL2020 and NUTmean, which represent the impacts of initial warm water, strong wind, and initial low nitrate concentration, respectively, on the marine ecosystem.

Figure 5 presents additional evidence highlighting the role of stratification in affecting the phytoplankton biomass, as shown by the differences between ALL2020 and WWTmean (2020 warm winter water effect), ALL2020 and ATMmean (2020 atmospheric forcing effect), and ALL2020 and NUTmean (2020 initial nitrate concentration effect). In the ALL2020–WWTmean and the ALL2020–ATMmean cases, the water temperature showed anomalies of 0.9 °C and − 2.5 °C at the surface and 1.1 °C and 0.6 °C at the bottom, respectively, by the end of May (Fig. 5a, b), implying that both warm initial temperature and strong winds caused a relative surface cooling anomaly and weakened the stability in the vertical water column^16^. This weakened stratification from mid-April could disrupt phytoplankton and nitrate concentration increase in the euphotic layer; therefore, the unfavorable conditions in the Yellow Sea in 2020 led to a decrease in spring phytoplankton biomass along with an increase in unused nitrate concentration (Fig. 5d, e, g, h). The finding aligns with many previous studies emphasizing the crucial role of vertical stratification, which creates an optimal depth for phytoplankton photosynthesis, in determining the magnitude and timing of phytoplankton bloom^5,11–13,28–30^. In contrast, the differences in temperature, chlorophyll-a concentration, and nitrate level between ALL2020 and NUTmean were feeble (Fig. 5c, f, i), indicating a negligible impact of nutrient concentrations on the 2020 spring bloom. In other words, the suppressed phytoplankton bloom in the Yellow Sea in 2020 was largely attributed to the weak stratification caused by strong winds and warm water temperatures, rather than a decrease in nutrient level during the COVID-19 pandemic.

## Summary and discussion

The spring bloom intensity in 2020 decreased by approximately 30% compared to previous years^15,16^, with the maximum chlorophyll-a concentration reaching only 50% of that observed in 2017–2019. Two previous studies have proposed reduced nutrient level^15^ and weakened thermal stratification from anomalous cold surface^16^ as possible causes of the change in phytoplankton biomass. To further support the findings of our previous research^16^, this subsequent study used observed S-ORS and marine physical-biogeochemical model data to investigate the critical factors contributing more to the weakened spring bloom in the Yellow Sea in 2020. In spring 2020 compared to the 2017–2019 period, the weakened stratification in the northeastern Yellow Sea, rather than the decreased nitrate concentration, plays an important role in suppressing phytoplankton biomass. This thermal structure creates unfavorable conditions for the photosynthesis of marine phytoplankton into the euphotic zone, reducing phytoplankton populations in the spring of 2020. Meanwhile, nitrate and phosphate concentrations in the northeastern Yellow Sea slightly decreased during April 2020 compared to the 2017–2019 average; these reductions were within the range of interannual variability observed in the long-term KODC data. Despite the significant reduction in PM_2.5_ level in March 2020 related to the COVID-19 pandemic, the nitrate concentration in early April 2020 showed a minor difference compared to the 2017–2019 period, thus having a marginal impact on the phytoplankton biomass in the spring of 2020.

When the spring stratification weakened in 2020, not only the phytoplankton biomass but also its community structure notably changed. One of the teams working together at the S-ORS conducted a chemotaxonomic analysis, a widely used approach for classifying phytoplankton groups, and elucidated distinct differences in the phytoplankton community between 2018–2019 and 2020 at the S-ORS (in a forthcoming paper). From late March to mid-April in both 2018 and 2019, Bacillariophyceae and Cryptophyceae groups were abundant throughout the entire water column. As the upper water temperature increased and the pycnocline developed, the phytoplankton bloom peaked in late April, with Chlorophytes and Cryptophyceae dominating the surface layer. However, in 2020, because of the weakened and delayed formation of the thermocline caused by a cooling surface anomaly^16^, the peak of the surface Chlorophytes bloom was absent. Instead, Bacillariophyceae and Cryptophyceae continued to dominate the entire water column until mid-May. These species seem to have a greater ability to thrive and withstand stronger vertical mixing compared to Chlorophytes.

According to our previous studies^16,20^, the weakened thermal stratification and anomalously cold water in the upper layer in the Yellow Sea in April 2020 are attributed to the enhanced latent heat loss from the ocean, mainly driven by the prevailing northerly winds under the extremely cold environmental conditions. In the future climate, these extreme cold events are projected to increase in intensity due to dynamic factors such as sea ice loss, snow cover reduction and warmer temperatures near the Arctic. This could facilitate the intrusion of cold air masses into the mid-latitudes^31,32^, leading to exceptional latent heat release; consequently, the vertical stratification in the Yellow Sea may be weaker. Such changes in stratification could hinder the spring phytoplankton bloom, as observed in the 2020 case. Therefore, a better understanding of the marine physical changes in the Yellow Sea will help mitigate potential damage to future marine phytoplankton biomass.

## Methods

### In-situ observational data

We used daily observations for 7 weeks during April to May in 2017–2020 on the S-ORS platform^33^, located in the northeastern Yellow Sea, approximately 50 km from the Korean peninsula 37°25′23.3ʺN, 124°44′16.9ʺE (Fig. 1): water temperature, fluorescence, and PM_2.5_, chlorophyll-a, and nutrient (nitrate and phosphate) concentrations. The vertical temperature was profiled twice a day using the SBE 19 plus v2 Conductivity-Temperature-Depth. The PM_2.5_ concentration was monitored using a Continuous Ambient Particulate Monitor (FH62C14, Thermo Fisher Scientific, Waltham, MA, USA) located on the roof deck of the S-ORS. Chlorophyll-a and nutrients concentration, and fluorescence profiling were performed once a day using seawater samples collected with the SBE 19 plus v2 Conductivity-Temperature-Depth, which was equipped with a 6 × 4-L Niskin bottle rosette sampler and the Wet Labs Eco Fluorometer, through the following filtration methods: (1) chlorophyll-a concentration: 0.5 L seawater samples were filtered through 25-mm GF/F filters, placed in a 15 mL conical tube containing 6 mL of 95% acetone, and extracted at 4 °C for 24 h in the dark. After filtration through a polytetrafluoroethylene filter, the chlorophyll-a concentration was determined using a Turner 10AU fluorometer (Turner Designs, San Jose, CA, USA)^34^. (2) nutrient concentration: seawater samples filtered through GF/F filters were frozen in 15 mL conical tubes in the dark until laboratory analysis. The filtered samples were analyzed in the laboratory using a Smartchem 200 (Smartchem 200, AMS Alliance, Italy). Vertical stratification was defined as the difference in water temperature between the surface (0 m) and bottom (40 m) levels. Phytoplankton variables (chlorophyll-a concentration and fluorescence) were integrated within the euphotic zone from the sea surface down to a depth of 20 m, relevant to active photosynthesis in marine phytoplankton.

To assess the long-term variability of nutrient concentrations, this study used the monthly nitrate and phosphate concentrations data for April 1994–2020 at eight stations in the central Yellow Sea from the KODC: station numbers 7 (125°E) and 9 (124.58°E) of lines 307 (36.925°N), 308 (36.33°N), 309 (35.855°N), and 310 (35.335°N) (see Fig. 1). The KODC was observed approximately every other month from February to December each year.

### Model configuration

To examine the chlorophyll-a concentration in response to the prescribed marine physical and chemical factors, we utilized GOTM-ERSEM, a one-dimensional turbulent mixing model coupled with a lower trophic level marine ecosystem model^35–37^. The GOTM is suitable for describing the simple thermal response to the initial temperature, atmospheric forcing, and turbulent processes associated with tides, wind, and waves without considering oceanic heat advection. Kim et al.^16^ successfully simulated spring temperature evolution observed at S-ORS using the GOTM, thus having a marginal thermal impact from oceanic advection associated with the Yellow Sea Warm Current and cold coastal current^38^. As followed by Kim et al.^16^ GOTM setting, the 1-D water column model has 101 vertical levels extending from the surface to 50 m and is based on a k-epsilon turbulence closure scheme for vertical turbulent mixing^39^ and the Oregon State University TPX09 tidal inversion software for tidal generation^40^. A marine food web model, ERSEM, is composed of major ecosystem types and biogeochemical components: four phytoplankton functional types, three zooplankton functional types, one heterotrophic bacterial functional type, particulate and dissolved organic matter, nutrients (nitrate, ammonium, phosphate, silicate, and iron), oxygen, and dissolved inorganic carbon. In particular, ERSEM has the advantage of simulating the important phytoplankton groups, microphytoplankton (dinoflagellates) and diatoms, in the Yellow Sea during spring^10,28,41,42^. More detailed information on the GOTM-ERSEM can be found in the reports by Allen et al.^35^ and Butenschön et al.^36^.

### Experimental design

The coupled GOTM-ERSEM experiments were initialized using water temperature and nitrate profiles obtained from the monitored S-ORS data. An observed salinity profile was employed to accurately represent the marine environment, in contrast to using a fixed initial salinity value of 32.36, as performed in our previous study^16^, although the water temperature primarily governs the water density in the Yellow Sea except during the summer monsoon period^14^. Meteorological background fields, including air pressure, air temperature, relative humidity, shortwave radiation, and zonal and meridional winds, were obtained from the European Centre for Medium-Range Weather Forecasts Reanalysis 5 (ERA5)^43^. Four experiments were conducted from 1 April to 20 May, with a time step of 1 h: ALL2020, WWTmean, ATMmean, and NUTmean (Table 1). ALL2020 served as a control run, representing the spring 2020 environment. It prescribed an initial water temperature of 7.6 °C, initial nitrate concentration of 7.3 μmol L^−1^, and atmospheric fields from 1 April to 20 May 2020. The WWTmean experiment used the same atmospheric boundary and initial nitrate conditions as ALL2020, except for an initial water temperature of 5.6 °C on average of 1 April 2017–2019, with. ATMmean and NUTmean also simulated normal atmospheric and nitrate (8.3 μmol L^−1^) conditions, respectively, instead of 2020 conditions (that is, ALL2020). The effects of the initial warm water temperature, strong cold winds, and initial lower nitrate concentration on the spring bloom in the Yellow Sea were estimated based on the differences between ALL2020 and WWTmean, ALL2020 and ATMmean, and ALL2020 and NUTmean, respectively.

### Supplementary Information

Supplementary Figure S1.

## Data Availability

All data are available from the following repositories: S-ORS data from the Korea Ocean Research Station project website https://kors.kiost.ac.kr and the OceanSITES network website https://dods.ndbc.noaa.gov/thredds/catalog/oceansites/catalog.html; KODC data from http://www.nfrdi.re.kr; and the ERA5 reanalysis data from https://cds.climate.copernicus.eu. The GOTM and ERSEM codes are available at https://gotm.net/portfolio/software/ and https://pml.ac.uk/Modelling_at_PML/Access_Code, respectively. The simulated GOTM-ERSEM data will be available from the authors upon reasonable request.
