# Supplementary material for: Impact of vertical stratification on the 2020 spring bloom in the Yellow Sea
Source: Sci Rep. 2023 Aug 31;13:14320. doi: 10.1038/s41598-023-40503-z (PMC10471739; doi:10.1038/s41598-023-40503-z)
Supplement: Supplementary file 1 — Supplementary Figure S1. [file 41598_2023_40503_MOESM1_ESM.docx]

**Supplementary Information**

**Impact of vertical stratification on the 2020 spring bloom in the Yellow Sea**

Go-Un Kim^1^, Jaeik Lee^1^, Yong Sun Kim^1,2^, Jae Hoon Noh^1^, Young Shin Kwon^1^, Howon Lee^1^, Meehye Lee^3^, Jongmin Jeong^1^, Myung Jin Hyun^1,4^, Jongseok Won^1,2^, and Jin-Yong Jeong^1*^

^1^Korea Institute of Ocean Science and Technology, Busan, South Korea

^2^Ocean Science and Technology School, Korea Maritime and Ocean University, Busan, South Korea

^3^Department of Earth and Environmental Sciences, Korea University, Seoul, South Korea

^4^Department of Ocean Science, University of Science and Technology, Daejeon, South Korea

*Corresponding author: Dr. Jin-Yong Jeong (jyjeong@kiost.ac.kr)


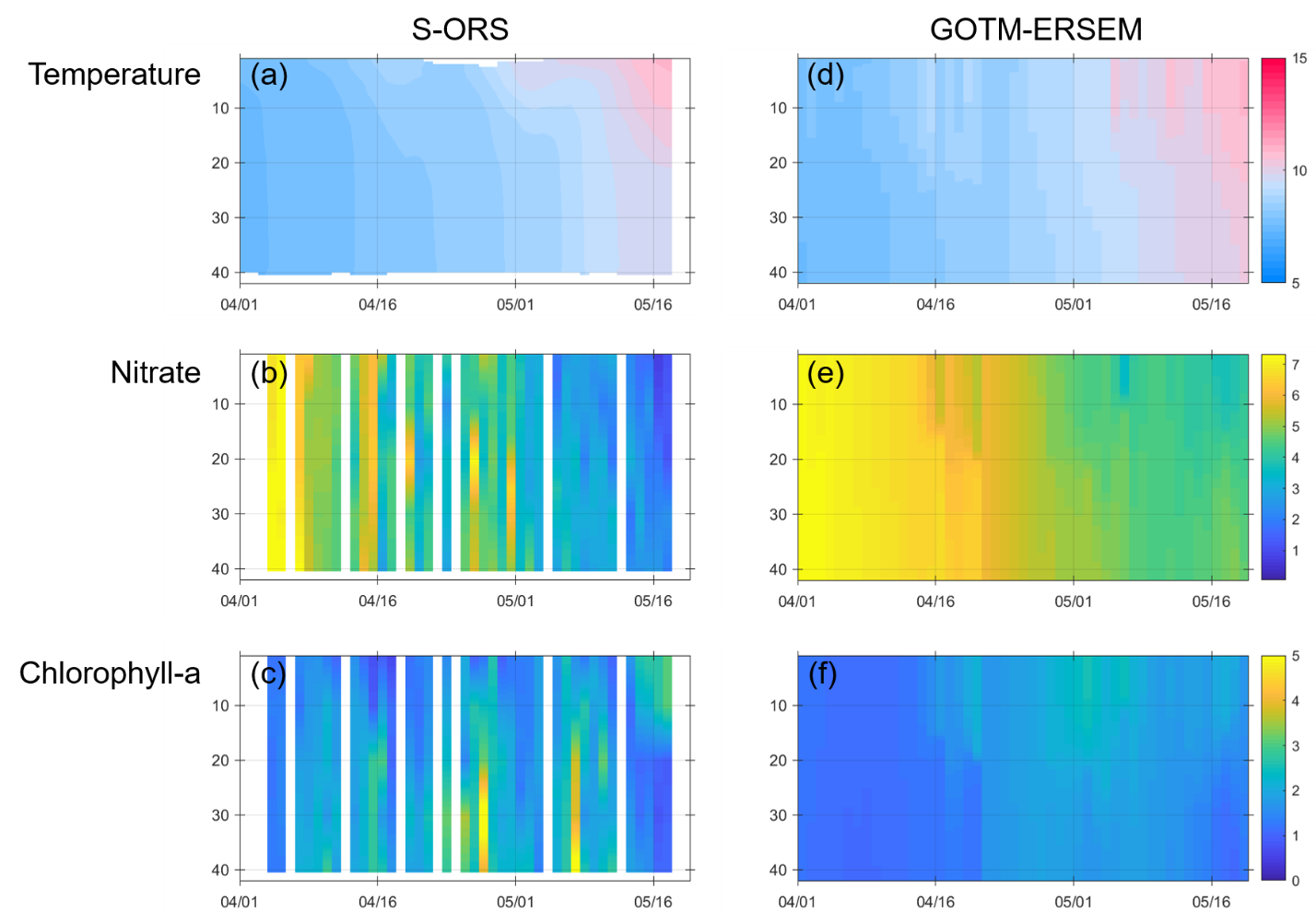


**Supplementary Figure S1. Comparison observation and numerical model data.** The time-depth distribution of (a, d) temperature (°C), (b, e) nitrate (μmol L^-1^), and (c, f) chlorophyll-a concentration (mg m^-3^) in the S-ORS and ALL2020 experiment.
